# Cumulative Update of a Systematic Overview Evaluating Interventions Addressing Polypharmacy

**DOI:** 10.1001/jamanetworkopen.2023.50963

**Published:** 2024-01-10

**Authors:** Michelle S. Keller, Nabeel Qureshi, Allison M. Mays, Catherine A. Sarkisian, Joshua M. Pevnick

**Affiliations:** 1 Division of General Internal Medicine, Department of Medicine, Cedars-Sinai Medical Center, Los Angeles, California; 2 Department of Health Policy and Management, Fielding School of Public Health, University of California, Los Angeles; 3 Division of Informatics, Department of Biomedical Sciences, Cedars-Sinai Medical Center, Los Angeles, California; 4 RAND Corporation, Los Angeles, California; 5 Section of Geriatrics, Department of Medicine, Cedars-Sinai Medical Center, Los Angeles, California; 6 Division of General Internal Medicine, Department of Medicine, David Geffen School of Medicine, University of California, Los Angeles; 7 VA Greater Los Angeles Healthcare System, Geriatrics Research Education & Clinical Center

## Abstract

**Importance:**

Polypharmacy is associated with mortality, falls, hospitalizations, and functional and cognitive decline. The study of polypharmacy-related interventions has increased substantially, prompting the need for an updated, more focused systematic overview.

**Objective:**

To systematically evaluate and summarize evidence across multiple systematic reviews (SRs) examining interventions addressing polypharmacy.

**Evidence Review:**

A search was conducted of MEDLINE, the Cochrane Database of Systematic Reviews, and the Database of Abstracts of Reviews of Effects for articles published from January 2017-October 2022, as well as those identified in a previous overview (January 2004-February 2017). Systematic reviews were included regardless of study design, setting, or outcome. The evidence was summarized by 4 categories: (1) medication-related process outcomes (eg, potentially inappropriate medication [PIM] and potential prescribing omission reductions), (2) clinical and functional outcomes, (3) health care use and economic outcomes, and (4) acceptability of the intervention.

**Findings:**

Fourteen SRs were identified (3 from the previous overview), 7 of which included meta-analyses, representing 179 unique published studies. Nine SRs examined medication-related process outcomes (low to very low evidence quality). Systematic reviews using pooled analyses found significant reductions in the number of PIMs, potential prescribing omissions, and total number of medications, and improvements in medication appropriateness. Twelve SRs examined clinical and functional outcomes (very low to moderate evidence quality). Five SRs examined mortality; all mortality meta-analyses were null, but studies with longer follow-up periods found greater reductions in mortality. Five SRs examined falls incidence; results were predominantly null save for a meta-analysis in which PIMs were discontinued. Of the 8 SRs examining quality of life, most (7) found predominantly null effects. Ten SRs examined hospitalizations and readmissions (very low to moderate evidence quality) and 4 examined emergency department visits (very low to low evidence quality). One SR found significant reductions in hospitalizations and readmissions among higher-intensity medication reviews with face-to-face patient components. Another meta-analysis found a null effect. Of the 7 SRs without meta-analyses for hospitalizations and readmissions, all had predominantly null results. Two of 4 SRs found reductions in emergency department visits. Two SRs examined acceptability (very low evidence quality), finding wide variation in the adoption of polypharmacy-related interventions.

**Conclusions and Relevance:**

This updated systematic overview noted little evidence of an association between polypharmacy-related interventions and reduced important clinical and health care use outcomes. More evidence is needed regarding which interventions are most useful and which populations would benefit most.

## Key Points

**Question:**

What is the current evidence base for interventions focused on addressing polypharmacy on process, clinical, and health care use outcomes?

**Findings:**

This systematic overview of 14 systematic reviews noted that interventions to address polypharmacy appeared to reduce potentially inappropriate prescribing omissions and commissions (low to very low evidence quality). However, these interventions did not appear to meaningfully reduce mortality and health care (moderate to very low evidence quality), falls (moderate to very low evidence quality), or quality of life (very low evidence quality).

**Meaning:**

The findings of this study suggest there remains a lack of high-quality evidence to support the broad implementation of polypharmacy-related interventions.

## Teaser

This systematic overview examines meta-analyses and systematic reviews on the use of polypharmacy-related interventions in adults.

## Introduction

The increase in the type and number of medications available to treat chronic and acute conditions has improved global longevity and quality of life. Yet this progress has also seen a corresponding increase in medication-related adverse events, often linked to increased polypharmacy,^1^ commonly defined as the regular use of 5 or more medications. In the US, rates of polypharmacy are estimated to be as high as 65% for adults aged 65 years and older.^2^ In Europe, estimates of the prevalence of polypharmacy in older adults range from 26% to 40%^3^; studies in other countries have found similar rates, from 36% in Australia^4^ to 42% in South Korea^5^ to 49% in India.^6^ The number of medications used on a regular basis has been found to independently be associated with mortality, falls, fractures, hospitalizations, and functional and cognitive decline.^1,7^

In recognition of the pressing issue of potentially harmful polypharmacy, in 2017, the World Health Organization (WHO) launched Medication Without Harms, a challenge focusing on polypharmacy, high-risk situations (eg, medication errors), and transitions of care.^8^ The goal of the WHO initiative was to reduce severe, avoidable, medication-related harm by 50% over 5 years. The WHO’s initiative mirrors efforts around the world to recognize, prevent, and address potentially harmful polypharmacy. The amount of literature regarding polypharmacy has correspondingly increased substantially. In our literature search, we found that, in 2017, researchers published approximately 5000 publications mentioning polypharmacy and polypharmacy-related interventions—only a slight increase from 2016. By 2021, there were 10 000 publications mentioning polypharmacy. In 2019, Anderson et al^9^ published a systematic overview of systematic reviews (SRs) of interventions aimed at addressing polypharmacy. The review, spanning from 2004 to 2017, identified 6 high-quality SRs meeting the criteria. It reported that polypharmacy interventions appeared to improve medication appropriateness but lacked consistent evidence for meaningful outcomes in health care use or mortality. Subsequently, we identified that 110 studies and 34 related SRs on polypharmacy have been published and indexed in PubMed since then. Given the increased focus and the need for updated research search, we present a cumulative overview of interventions addressing polypharmacy. We aimed to ascertain whether recent evidence strengthens the case for improved outcomes, such as quality of life, falls, cognitive and physical function, health care costs, and health care use and mortality outcomes.

## Methods

Reporting for this study is based on the Preferred Reporting Items for Systematic Reviews and Meta-analyses (PRISMA) reporting guideline and other available methodological guidance for systematic overviews.^10^

### Search Methods

MEDLINE, the Cochrane Database of Systematic Reviews, and the Database of Abstracts of Reviews of Effects were searched for articles published from January 2017 to October 2022, as well as those identified in a previous overview (January 2004 to February 2017). eAppendix 1 in Supplement 1 provides the full search strategy.

### Selection of SRs

Systematic reviews, with or without meta-analyses, were eligible for review if they evaluated interventions addressing polypharmacy in adults (age ≥18 years). eAppendix 2 in Supplement 1 provides the full selection strategy. Polypharmacy-related interventions could include any or all of the following: administering type I (review of the prescription list), type II (review of prescriptions and assessing for adherence), or type III (include the former 2 and address issues relating to the patient’s use of medications in the context of their diagnoses) medication reviews; deprescribing, which involves the process of systematically stopping or reducing the dose of medications; conducting patient education and counseling; holding case conferences with interdisciplinary teams; identifying potentially inappropriate medications (PIMs) or potential prescribing omissions (PPOs); using pharmacogenomics to determine whether individual differences in the expression of a protein or enzyme affect the metabolism of a drug; using health care professional education and clinical decision support; simplifying medication regimens to reduce complexity and improve adherence; using guidelines or tools, such as the American Geriatrics Society Beers Criteria or Screening Tool of Older Persons’ Potentially Inappropriate Prescriptions (STOPP)/Screening Tool^11^; and using medication management tools, such as pill organizers or smartphone apps, to assist patients with using their medications correctly.^9,12,13^

### Data Collection

Following our pilot data collection, we gathered titles from search sources. Two independent reviewers (M.S.K. and N.Q.) assessed these titles, resolving disagreements through discussion. We refined our inclusion criteria and proceeded to review abstracts and full texts. Reviewers resolved discrepancies by reaching a consensus during meetings.

### Evaluation of SR Quality

We assessed the methodologic quality of each relevant SR using a previously tested tool with demonstrated validity. We used the A Measurement Tool to Assess Systematic Reviews (AMSTAR 2) instrument.^14^ AMSTAR 2 has 16 requisite items that are rated as present or absent, such that each SR may receive a score ranging from 0 to 16.

### Evaluation of the Quality of Evidence

We used the Grading of Recommendations, Assessment, Development, and Evaluations (GRADE) framework, which can be applied to a body of evidence across outcomes.^15^ The framework criteria include study design, study quality, consistency, generalizability, and publication bias.

### Data Extraction and Synthesis

Two researchers (M.S.K. and N.Q.) extracted the data into a standardized abstraction spreadsheet (eAppendix 3 in Supplement 1), which included elements of the AMSTAR 2 and GRADE criteria.^14,16,17^ We grouped outcomes reported in the SRs into 4 categories: (1) medication-related process outcomes (eg, reduction in PIMs or PPOs, increase in medication appropriateness or medication adherence), (2) clinical and functional outcomes, (3) health care use and economic outcomes, and (4) acceptability of the intervention among patients and clinicians. We did not conduct pooled analyses given the relatively small number of meta-analyses findings reported for each outcome type.

## Results

### Study Selection

Our search strategy resulted in 287 titles for review, which were narrowed to 84 abstracts. We further narrowed this to 28 full-text articles and 6 SRs from the previous systematic overview. Our final count was 14 SRs, including 11 SRs^18,19,20,21,22,23,24,25,26,27,28^ from our current search and 3 SRs from the previous systematic overview (Figure, Table 1). An older Cochrane review from the previous overview was excluded because we had an updated review,^31^ and 2 SRs from the previous overview were excluded because they did not fit our updated, more restrictive inclusion criteria.^32,33^

**Figure.**
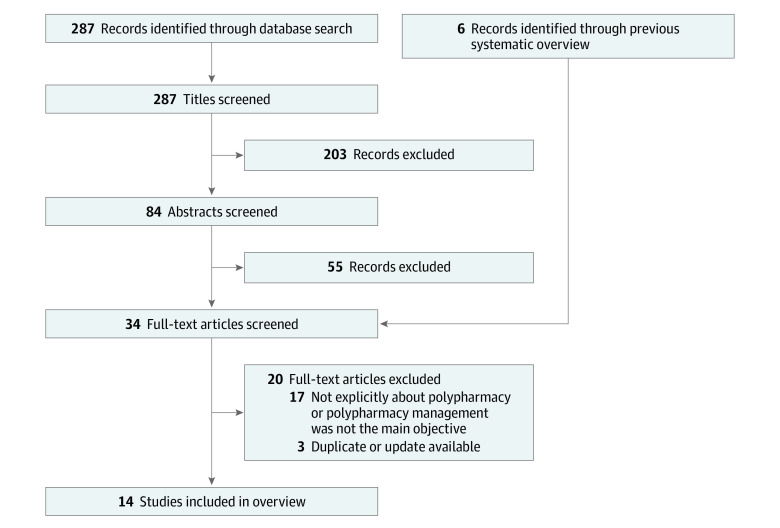
Flowchart of Included Studies

**Table 1.** Summary of Included Systematic Reviews Aimed at Addressing Polypharmacy

Source	Literature search coverage	No. of articles included and study designs	Population	Setting	Intervention type	Primary outcome measure	Major conclusion
Johansson et al,^29^ 2016	Database inception-July 2015	25 Studies (21 RCTs, 4 non-RCTs)	Adults aged ≥65 y (or 80% of study population aged ≥65 y) receiving ≥4 medications	Community settings/primary care (15), care/nursing homes or assisted living facilities (7), hospital (3)	Pharmacist-led medication review or multicomponent intervention (13), physician-led intervention (4), multidisciplinary team-led intervention (8)	Mortality, hospitalization, changes in No. of medications	The intervention strategies evaluated did not present compelling evidence on reducing mortality, hospitalization rates, or medication use. The interventions in the studies are complex and it is difficult to assess which intervention should be implemented to reduce inappropriate polypharmacy.
Page et al,^30^ 2016	Database inception-February 2015	116 Studies (56 RCTs, 22 comparative studies with concurrent control, and 37 comparative studies without concurrent control) (1 article reported on 2 studies)	Older adults (age ≥65 y) receiving ≥1 medication	Community settings (73), residential aged care facilities (29), hospital (14), hospital/community (1), community/residential care (1)	Deprescribing of ≥1 medication, medication class, or therapeutic category; deprescribing polypharmacy; medication reviews; educational programs for health care professionals	Mortality	A notable reduction in mortality was observed in nonrandomized studies and those focusing on patient-specific interventions; however, there was no statistically significant decrease in mortality in randomized studies and studies centered on generalized education programs.
Thillainadesan et al,^18^ 2018	January 1996- April 2017	9 RCTs	Older populations with a median age of ≥65 y	Hospital (9)	Deprescribing interventions were either pharmacist- (n = 4), physician- (n = 4), or multidisciplinary team- (n = 1) led	Reduction in PIMs	Based on the available evidence, it appears that deprescribing interventions within a hospital setting are viable and, in most cases, may reduce PIMs while also maintaining safety.
Rankin et al,^13^ 2018	Database inception-February 2018 (18 studies before 2013 were from a prior review)	18 RCTs, 10 cluster RCTs, 2 non-RCT studies, 2 controlled pre-post studies	Older adults (age ≥65 y, or 80% of study population aged ≥65 y) who had >1 long-term medical condition and were receiving polypharmacy (receiving ≥4 regularly prescribed medications)	Hospital (16), primary care (10), nursing homes (6)	Complex, multifaceted interventions (31), physician-led deprescribing (4), educational interventions to prescribers (10)	Medication appropriateness, PIMs, PPOs, hospital admissions	The outcomes of interventions aimed at enhancing appropriate polypharmacy, such as medication reviews, on clinically significant improvement remain uncertain. Nevertheless, these interventions may offer some slight benefits in terms of reducing PPOs. This conclusion is drawn from just 2 studies, both of which had substantial limitations concerning bias risk.
Mizokami et al,^19^ 2019	January 1972-March 2017	9 RCTs	Older adults receiving ≥5 medications	Hospital inpatients (3), community outpatients (6)	CMR of 3 levels: type I (prescription review), type II (medication adherence review), type III (focus on face-to face review of medicines and condition with the patient)	Total number of unplanned hospitalizations or rehospitalizations, and the number of patients experiencing (single or multiple) hospitalizations or rehospitalizations between CMR types	Type III CMRs led to a significant decrease in unplanned admissions among elderly patients during their hospitalization, but had no association with hospital admission rates for outpatient older adults.
Ali et al,^20^ 2020	Database inception-April 2019	7 RCTs, 2 observational studies	Older adults aged ≥65 y with chronic conditions who were receiving ≥5 medications/d	Community settings (6), residential care facilities (2), geriatric outpatient clinics (1)	Medication reviews (4), geriatric assessments/ medication screening (2) deprescribing interventions (3)	Falls, physical function	The evidence indicates that interventions addressing polypharmacy have favorable and clinically significant impacts on mobility outcomes, as evidenced by a decrease in the incidence of falls.
Lum et al,^21^ 2020	Database inception-May 2019	5 RCTs, 1 retrospective quasi-experimental study	Adults patients (age ≥18 y) with multiple prescription medications and having ≥1 of 3 cardiometabolic diseases: stroke, heart disease, and type 2 diabetes	Cardiac rehabilitation clinic (1), community setting (5)	Pharmacist-led multicomponent intervention (5), clinical-decision support tool–based intervention (1)	Quality of life, drug-related problems, surrogate markers, health care use, and costs	The outcomes of polypharmacy interventions for individuals with cardiometabolic diseases are inconsistent. Interventions involving more frequent and longer direct patient care sessions might yield the most favorable outcomes.
Hasan Ibrahim et al,^22^ 2021	Database inception-April 2020	6 RCTs, 1 CBA	Older adults (age ≥65 y) with both multimorbidity (presence of ≥2 long-term conditions) and polypharmacy (concomitant use of ≥4 medications)	Primary care (7)	Practice-based pharmacist-led interventions in primary care (7)	Drug-related problems, medication appropriateness, medication adherence, No. of medications, quality of life, hospital admissions/ readmissions	This systematic review revealed a scarcity of evidence, with only 7 studies exploring the services provided by pharmacists in optimizing medication management for older individuals dealing with both multimorbidity and polypharmacy.
Laberge et al,^23^ 2021	2004-2020	6 cluster RCTs, 3 RCTs, 1 pre-post, 1 cohort with control	Populations at least 80% older adults (age ≥65 y) with multimorbidity (defined as having ≥2 chronic conditions) and having polypharmacy	Primary care clinics (n = 4), nursing homes (n = 1) pharmacies (n = 3), hospital (1), academic medical center (1)	CMRs (10), pharmacogenetic testing with a clinical decision-support tool (1)	PIM use, ADEs, No. of medications, hospital admissions/ readmissions, costs of interventions, cost per PIM avoided, No. of PIMs avoided, No. of ADEs avoided	Because of the diversity in reported outcomes and the suboptimal quality of the economic assessments, the authors acknowledged their inability to reach a definite conclusion regarding the cost-effectiveness of interventions aimed at optimizing medication use.
Lee et al,^24^ 2021	Database inception-August 2020	3 RCTs, 2 cluster RCTs	Older adults (age ≥65 y)	Community setting (4), long-term care (1)	Pharmacist-led FRID deprescribing appropriateness (3), physician-led FRID deprescribing appropriateness (2)	Rate of falls, incidence of falls, rate of fall-related injuries	Insufficient strong and high-quality evidence is available to either support or contradict the association between a deprescribing strategy for fall-related injuries in older adults solely based on FRID
Tasai et al,^25^ 2021	Database inception-January 2018	4 RCTs (1 study not included in the quantitative analysis due to lack of data)	Older adults (age ≥65 y) receiving ≥4 prescribed medications	Community settings (4)	CMR (4)	Quality of life, hospitalizations, ED visits, medication adherence	Evidence illustrates that comprehensive clinical medication reviews conducted by community pharmacists for older individuals with multiple medications may help in reducing the risk of ED visits.
O’Shea et al,^26^2022	Not reported	3 RCTs; 6 noncomparative studies, 3 observational studies	Adults with multimorbidity and polypharmacy	Community settings (10), long-term care (2),	Pharmacist-led CMR with pharmacogenetics testing (8), physician-led pharmacogenetics testing (3), pharmacogenetics review (1)	Hospitalizations, ED visits, outpatient visits, health care costs, costs of genetic testing	Owing to the absence of methodologically strong, high-quality research, limited sample sizes, and relatively brief follow-up periods, the authors encountered limited evidence regarding pharmacogenetic interventions in enhancing outcomes for patients with both multimorbidity and prescribed polypharmacy.
Reeve et al,^27^ 2022	2009-June 2020	7 RCTs, 2 non-RCTs, 4 pre-post studies, 2 prospective cohort, 2 retrospective cohort, 2 cross-sectional, 1 exploratory study	Older adults (age ≥50 y) with ≥2 long-term medical conditions and polypharmacy (≥5 long-term medications/d)	Primary care (10), secondary care (7), tertiary care (2), pharmacy call center (1)	Physician-led deprescribing (11), pharmacist-led deprescribing (5), multi-disciplinary team deprescribing (4)	Associations (eg, prescribing-related outcomes), associations (eg, hospitalizations, falls, quality of life), safety (eg, adverse events), acceptability (eg, satisfaction)	The collective reviews acknowledge that deprescribing is a multifaceted intervention and offer endorsement for the safety of well-structured deprescribing approaches. However, the authors also emphasize the necessity of incorporating patient-centered and contextual elements into the best practice models. The authors concluded that the studies provided clear accounts of the objectives of the deprescribing interventions and the target patient populations. Nevertheless, they frequently lacked comprehensive information concerning the individuals responsible for delivering the intervention and the specific methods used.
Stötzner et al,^28^ 2022	Database inception-July 2021	15 RCTs, 27 non-comparison pre-post studies, 5 randomized pre-post approach, 9 retrospective studies, 2 study designs not reported	Patients with all psychiatric diagnoses	Nursing homes (39), psychiatric inpatient settings (10), psychiatric outpatient settings (9)	Individual medication review (22); CMR (36); educational programs, guideline reviews, or consulting services (12); automatic alerts (4)	Drug-related problems, medication appropriateness, hospital admissions, ED visits, falls, frailty measures, mortality, cognitive status, health care costs	Interventions targeting polypharmacy can result in enhanced drug-related outcomes among psychiatric populations. Nevertheless, changes in clinical outcomes were frequently minimal and typically less reported.

Abbreviations: ADE, adverse drug event; CBA, cost-benefit analysis; CMR, comprehensive medication review; ED, emergency department; FRID, fall risk–increasing drugs; PIM, potentially inappropriate medication; PPO, potential prescribing omission; RCT, randomized clinical trial.

### Study Characteristics

Included SRs were published between 2016 and 2022; studies from before 2017 were from the previously published systematic overview.^9^ Seven SRs included meta-analyses,^13,19,20,24,25,29,30^ 10 SRs included both observational studies and randomized clinical trials,^13,20,21,22,23,24,26,27,28,29^ and 4 SRs included only randomized clinical trials.^18,19,25,30^ Across all SRs, the mean (SD) AMSTAR 2 score was 10.8 (2.8) (of a possible of 16); across SRs with meta-analyses, the mean AMSTAR 2 score was 12.6 (2.8), and across SRs without meta-analyses, the mean was 9 (1.5) (eTable 1 in Supplement 1).

The SRs included in our review represented a total of 179 unique studies. Of these, 80% (143) were cited by only 1 SR, 12% (20) were cited by 2 SRs, 7% (12) were cited by 3 SRs, and 0.2% (4) were cited by 4 or more SRs (eTable 3 in Supplement 1). The number of studies included ranged from 7 to 58; the mean (SD) was 16 (15).

Of the 14 SRs, 10 focused exclusively on older adults, defined as a population aged 65 years or older. Another inclusion criterion in some SRs was the presence of multimorbidity or having at least 1 chronic disease.^21,22,23,26,27^ One study focused on patient populations with psychiatric diagnoses,^28^ while another focused on patient populations with cardiometabolic chronic diseases (ie, stroke, heart disease, or type 2 diabetes).^21^ Only 1 study included analyses of subpopulations separately: participants aged 80 years and older, participants aged 65 to 79 years, participants living with dementia, and participants who were cognitively intact.^30^

The SRs also included diverse study settings. Nearly all SRs included studies set in primary care, outpatient care, or community settings^13,19,20,21,22,23,24,25,26,27,28,29,30^; 1 focused solely on interventions set in the hospitals^18^; and 8 included studies set in nursing homes or other long-term care facilities.^13,20,23,24,28,29,30^ Only 1 SR synthesized results by setting, examining studies in nursing home settings, psychiatric inpatient settings, and psychiatric outpatient settings separately.^28^

In nearly all studies included in the SRs, medication reviews formed the base of the intervention. Studies detailed in the SRs included numerous other elements, including pharmacogenetic testing,^26^ physician- or patient-focused educational programs, guidelines or criteria (eg, Beers Criteria,^34,35^ STOPP/START^11,36^), tools based on guidelines (eg, Tool to Reduce Inappropriate Medication recommendations based on 2012 Beers Criteria and the Assessing Care of the Vulnerable Elderly tool to identify PPOs), consultancy services, multidisciplinary teams, home safety checklists, computerized clinical decision support, and geriatric assessments. Intervention types in each SR are detailed in eTable 2 in Supplement 1. In many studies detailed in the SRs, a pharmacist or multidisciplinary team reviewed the patient’s medications and provided recommendations or educational materials to prescribers.

### Medication-Related Process Outcomes

Nine SRs reported medication-related process outcomes (Table 2).^13,18,21,22,23,25,28,29,30^ These included total number of and changes in number of medications, total number of and changes in the number of PIMs, drug-related problems, medication-related adverse events, drug-gene interactions, drug-drug interactions, medication appropriateness, medication adherence, actionable pharmacogenetic recommendations, total number of PPOs, and recommendations for changes in medications.

**Table 2.** Medication-Related Process Outcomes From Systematic Reviews of Studies Examining Polypharmacy Interventions

Systematic review	Medication-related process outcomes					
	No. of medications	No. of PIMs	No. of PPOs	Medication appropriateness^a^	Medication-related problems^b^	Medication adherence
Johansson et al,^29^ 2016	No meta-analysis	No meta-analysis	NA	No meta-analysis	No meta-analysis	No meta-analysis
	DE	ME	NA	IE	ME	ME
	Evidence quality: very low	Evidence quality: very low	NA	Evidence quality: very low	Evidence quality: very low	Evidence quality: very low
Page et al,^30^ 2016	MD, −0.99 (95% CI, −1.83 to −0.14)	MD, −0.49 (95% CI, −0.70 to −0.28)	NA	NA	No meta-analysis	NA
	DE	DE	NA	NA	NE	NA
	Evidence quality: very low	Evidence quality: low	NA	NA	Evidence quality: very low	NA
Thillainadesan et al,^18^ 2018	NA	No meta-analysis	NA	No meta-analysis	No meta-analysis	NA
	NA	ME	NA	ME	ME	NA
	NA	Evidence quality: low	NA	Evidence quality: very low	Evidence quality: very low	NA
Rankin et al,^13^ 2018	NA	No. of PIMs: SMD, −0.22 (95% CI, −0.38 to −0.05)	No. of PPOs: SMD, −0.81 (95% CI, −0.98 to −0.64)	MD, −4.76 (95% CI, −9.20 to −0.33)	No meta-analysis	No meta-analysis
	NA	DE	DE	IE	ME	ME
	NA	% of patients with at least 1 PIM: RR, 0.79 (95% CI, 0.61-1.02)	% of patients with a PPO: RR, 0.40 (95% CI, 0.18- 0.85)	NA	NA	NA
	NA	NE	DE	NA	NA	NA
	NA	Evidence quality: very low	Evidence quality: low	Evidence quality: very low	Evidence quality: very low	Evidence quality: very low
Lum et al,^21^2020	NA	NA	NA	No meta-analysis^c^	No meta-analysis	No meta-analysis^c^
	NA	NA	NA	IE	DE	IE
	NA	NA	NA	Evidence quality: very low	Evidence quality: very low	Evidence quality: very low
Hasan Ibrahim et al,^22^ 2021	No meta-analysis	NA	NA	No meta-analysis	No meta-analysis	No meta-analysis
	ME	NA	NA	IE	ME	ME
	Evidence quality: very low	NA	NA	Evidence quality: very low	Evidence quality: very low	Evidence quality: very low
Laberge et al,^23^ 2021	No meta-analysis	No meta-analysis	NA	NA	No meta-analysis	NA
	NE	DE	NA	NA	DE	NA
	Evidence quality: very low	Evidence quality: very low	NA	NA	Evidence quality: very low	NA
Tasai et al,^25^ 2021	NA	NA	NA	NA	NA	No meta-analysis^c^
	NA	NA	NA	NA	NA	IE
	NA	NA	NA	NA	NA	Evidence quality: very low
Stötzner et al,^28^ 2022	No meta-analysis	NA	No meta-analysis	No meta-analysis	No meta-analysis	NA
	ME	NA	DE	ME	DE	NA
	Evidence quality: low	NA	Evidence quality: very low	Evidence quality: low	Evidence quality: very low	NA

Abbreviations: DE, decreased effect; IE, increased effect; MD, mean difference; ME, mixed effect; NA, not applicable; NE, null effect; PIM, potentially inappropriate medication; PPO, potential prescribing omission; RR, risk ratio; SMD, standardized mean difference.

^a^ An increase in medication appropriateness is the preferred direction.

^b^ Adverse drug reaction, drug-drug interaction, and medication errors.

^c^ Results are from 1 study only.

#### Total Number of Medications

We identified 5 SRs that examined the interventions in terms of the total number of medications; only 1 SR conducted a pooled analysis, finding a reduction in the total number of medications (mean difference [MD], −0.99; 95% CI, −1.83 to −0.14).^30^ Another SR also found an overall reduction,^29^ 2 found mixed effects (ie, some studies in the SRs found reductions, others found null effects),^22,28^ and 1 found a null effect.^23^

#### Potentially Inappropriate Medications

Five studies examined the number of PIMs. Two SRs using pooled analyses found a significant reduction in the number of PIMs (standardized MD [SMD], −0.22; 95% CI, −0.38 to −0.05^13^; and MD, −0.49; 95% CI, −0.70 to −0.28),^30^ but 1 found no significant difference in the proportion of patients with at least 1 PIM (risk ratio [RR], 0.79; 95% CI, 0.61-1.02).^13^ Three SRs without meta-analyses or pooled analyses found mixed effects, with most studies identifying some reduction in PIMs, but others finding null effects.^18,23,29^

#### Potential Prescribing Omissions

Only 2 SRs examined PPOs: 1 found both a significant reduction in the number of PPOs (SMD, −0.81; 95% CI, −0.98 to −0.64) and a reduction in the proportion of patients with at least 1 PPO (RR, 0.40; 95% CI, 0.18-0.85).^13^ The other SR, which included only 1 study and no pooled analyses, also found a decrease.^28^

#### Medication Appropriateness

Medication appropriateness, often measured via the Medication Appropriateness Index,^37^ improved in 4^13,21,22,29^ of 6 SRs. The other 2 SRs^18,28^ found mixed effects.

#### Medication-Related Problems

Of the 8 SRs that examined medication-related problems, which include adverse drug reactions and drug-drug interactions, 3 SRs found a reduction,^21,23,28^ 4 found mixed effects,^13,18,22,29^and 1 SR^30^ found a null effect.

#### Medication Adherence

Five SRs measured medication adherence. Two of these found an increase in medication adherence,^21,25^ while the other 3 found mixed effects.^13,22,29^

### Clinical and Functional Outcomes

Twelve SRs reported clinical and functional outcomes (Table 3).^13,18,20,21,22,23,24,25,26,28,29,30^ These included mortality, frailty, delirium, cardiovascular events, severity of illness, depression, pain, anxiety, physical function, cognitive status, infections, falls, fall-related injuries, fractures, blood pressure, hemoglobin A_1c_, fasting blood glucose level, cholesterol level, and triglyceride level. Seven SRs reported quality-of-life outcomes.^13,18,21,22,25,28,30^

**Table 3.** Clinical and Functional Outcomes From Systematic Reviews of Studies Examining Polypharmacy Interventions

Systematic review	Clinical and functional outcomes					
	Mortality	Incidence of falls	Rate of falls	No. of falls	Quality of life	Cognitive or physical function
Johansson et al,^29^ 2016	All-cause mortality in all studies: OR, 1.02 (95% CI, 0.84-1.23)	NA	NA	NA	No meta-analysis	No meta-analysis
	NE	NA	NA	NA	NE	ME
	All-cause mortality in studies with longer follow-up periods (12-18 mo): OR, 0.93 (95% CI, 0.69-1.24)	NA	NA	NA	NA	NA
	NE	NA	NA	NA	NA	NA
	All-cause mortality in studies with shorter follow-up periods (2-6 mo): OR, 1.13 (95% CI, 0.86-1.50)	NA	NA	NA	NA	NA
	NE	NA	NA	NA	NA	NA
	All-cause mortality in RCTs or CRCTs: OR, 1.05 (95% CI, 0.85-1.29)	NA	NA	NA	NA	NA
	NE	NA	NA	NA	NA	NA
	Evidence quality: low	NA	NA	NA	Evidence quality: very low	Evidence quality: very low
Page et al,^30^ 2016	Mortality in randomized studies: OR, 0.82 (95% CI, 0.61-1.11)	Risk of experiencing at least 1 fall: OR, 0.65 (95% CI, 0.40-1.05)	NA	No. of falls among patients with at least 1 fall: MD, −0.11 (95% CI, −0.21 to −0.02)	No meta-analysis	No meta-analysis
	NE	NE	NA	DE	NE	NE
	Mortality in nonrandomized studies: OR, 0.32 (95% CI, 0.17-0.60)	NA	NA	NA	NA	NA
	DE	NA	NA	NA	NA	NA
	Mortality in participants aged ≥80 y (randomized studies only): OR, 0.88 (95% CI, 0.58-1.34)	NA	NA	NA	NA	NA
	NE	NA	NA	NA	NA	NA
	Mortality in participants aged 65-79 y (randomized studies only): OR, 0.64 (95% CI, 0.40-1.04)	NA	NA	NA	NA	NA
	NE	NA	NA	NA	NA	NA
	Mortality in participants living with dementia (randomized studies only): OR, 0.89 (95% CI, 0.63-1.27)	NA	NA	NA	NA	NA
	NE	NA	NA	NA	NA	NA
	Mortality in cognitively intact participants (randomized studies only): OR, 0.64 (95% CI, 0.36-1.13)	NA	NA	NA	NA	NA
	NE	NA	NA	NA	NA	NA
	Evidence quality: low	Evidence quality: very low	NA	Evidence quality: very low	Evidence quality: very low	NA
Thillainadesan et al,^18^ 2018	No meta-analysis	No meta-analysis	No meta-analysis^a^	No meta-analysis^a^	No meta-analysis	No meta-analysis
	NE	ME	DE	NE	ME	ME
	Evidence quality: very low	Evidence quality: very low	Evidence quality: very low	Evidence quality: very low	Evidence quality: very low	Evidence quality: very low
Rankin et al,^13^ 2018	NA	NA	NA	NA	No meta-analysis	NA
					ME	
					Evidence quality: very low	
Ali et al,^20^ 2020	NA	Incidence of falls (clinical trials only): RR, 0.87 (95% CI, 0.57-1.31)	NA	No meta-analysis^a^	NA	Physical function (clinical trials only): SMD, 0.00 (95% CI, −0.21 to 0.20)
	NA	NE	NA	NE	NA	NE
	NA	Incidence of falls in studies where medications were discontinued: RR, 0.51 (95% CI, 0.36-0.71)	NA	NE	NA	NA
	NA	DE	NA	NA	NA	NA
	NA	Evidence quality: low	NA	Evidence quality: moderate	NA	Evidence quality: low
Lum et al,^21^ 2020	NA	NA	NA	NA	No meta-analysis	NA
	NA	NA	NA	NA	ME	NA
	NA	NA	NA	NA	Evidence quality: very low	NA
Hasan Ibrahim et al,^22^ 2021	NA	NA	NA	NA	No meta-analysis	NA
	NA	NA	NA	NA	NE	NA
	NA	NA	NA	NA	Evidence quality: very low	NA
Laberge et al,^23^ 2021	NA	NA	NA	NA	NA	No meta-analysis^a^
	NA	NA	NA	NA	NA	NE
	NA	NA	NA	NA	NA	Evidence quality: very low
Lee et al,^24^ 2021	NA	Falls incidence: RR, 1.04 (95% CI, 0.86-1.26)	Falls rate: rate ratio, 0.98 (95% CI, 0.63-1.51)	NA	NA	NA
	NA	NE	NE	NA	NA	NA
	NA	Falls incidence: RD, 0.01 (95% CI, −0.06 to 0.09)	NA	NA	NA	NA
	NA	NE	NA	NA	NA	NA
	NA	Evidence quality: very low	Evidence quality: very low	NA	NA	NA
Tasai et al,^25^ 2021	NA	NA	NA	NA	No meta-analysis	NA
	NA	NA	NA	NA	ME	NA
	NA	NA	NA	NA	Evidence quality: very low	NA
O’Shea et al,^26^2022	No meta-analysis^a^	NA	NA	No meta-analysis^a^	NA	NA
	NE	NA	NA	NE	NA	NA
	Evidence quality: very low	NA	NA	Evidence quality: very low	NA	NA
Stötzner et al,^28^ 2022	No meta-analysis; evidence quality: very low	No meta-analysis	NA	No meta-analysis	No meta-analysis	No meta-analysis
	ME	DE	NA	DE	IE	ME
	Evidence quality: very low	Evidence quality: very low	NA	Evidence quality: very low	Evidence quality: very low	Evidence quality: very low

Abbreviations: CRCT, cluster randomized clinical trial; DE, decreased effect; IE, increased effect; MD, mean difference; ME, mixed effect; NA, not applicable; NE, null effect; OR, odds ratio; RD, risk difference; RR, risk ratio; SMD, standardized mean difference.

^a^ Results are from 1 study only.

#### Mortality

Five SRs examined the polypharmacy interventions in terms of mortality, of which 4 found a null effect^18,26,29,30^ and 1 found mixed effects.^28^ Two SRs included meta-analyses. One SR using numerous sensitivity analyses reported an odds ratio (OR) of 1.02 in all studies examining all-cause mortality (95% CI, 0.84-1.23),^29^ an OR of 0.93 (95% CI, 0.69-1.24) among studies with longer follow-up periods (12-18 months), an OR of 1.13 (95% CI, 0.86-1.50) among studies with shorter follow-up periods (2-6 months), and an OR of 1.05 (95% CI, 0.85-1.29) among randomized clinical trials. The other SR reported an OR of 0.82 (95% CI, 0.61-1.11) among randomized studies and an OR of 0.32 (95% CI, 0.17-0.60) among nonrandomized studies.^30^ The authors also conducted subpopulation analyses, finding a larger reduced effect of mortality associated with the interventions in participants aged 65 to 79 years (OR, 0.64; 95% CI, 0.40-1.04) vs participants aged 80 years or older (OR, 0.88; 95% CI, 0.58-1.34).^30^ The authors also found a larger effect size among patients without dementia (OR, 0.64; 95% CI, 0.36-1.13) compared with patients living with dementia (OR, 0.89; 95% CI, 0.63-1.27).^30^

#### Falls

Five SRs examined the incidence of falls: 2 of these found a reduction (1 SR only found a reduction when medications were discontinued),^20,28^ 1 found mixed effects (ie, a mix of null effects and small reductions in fall incidence),^18^and 2 found a null effect.^24,30^ Of the SRs that conducted pooled analyses for the incidence of falls, 1 found a nonsignificant RR of 0.87 (95% CI, 0.57-1.31), but a larger, significant outcome when the PIMs were discontinued (RR, 0.51; 95% CI, 0.36-0.71), using a fixed-effects meta-analysis.^20^ The authors found that the pooled effect was not significant when a random effects meta-analysis was used (RR, 0.54; 95% CI, 0.27-1.06).^20^ For the risk of experiencing at least 1 fall, 1 SR found an OR of 0.65 (95% CI, 0.40-1.05).^30^ Finally, with regard to fall risk incidence, another SR reported null effects, with a fall risk incidence of 1.04 (95% CI, 0.86-1.26) and a risk difference of 0.01 (95% CI, −0.06 to 0.09).^24^ Two SRs examined the rate of falls. One found a null effect (rate ratio, 0.98; 95% CI, 0.63-1.51),^24^ while the other did not conduct a pooled analysis, but found a reduction based on 1 study.^18^ Five SRs examined the number of falls. One SR conducted a pooled analysis of the number of falls among patients with at least 1 fall, finding an MD of −0.11 (95% CI, −0.21 to 0.02).^30^ Three other SRs relied on 1 study alone each to report findings; all found a null effect.^18,20,26^ Another SR did not conduct a meta-analysis, but found a reduction in the number of falls among studies examined.^28^

#### Quality of Life

Eight SRs examined quality of life; 1 of these found an increase among individuals receiving a polypharmacy intervention,^28^ 4 found mixed effects,^13,18,21,25^ and 3 found null effects.^22,29,30^ Six SRs examined cognitive or physical function; 3 of these found mixed effects^18,28,29^ and 3 found null effects.^20,23,30^ The only SR conducting a pooled analysis of physical function in clinical trials reported a null result (SMD, 0.00; 95% CI, −0.21 to 0.20).^20^

### Health Care Use and Economic Outcomes

Ten SRs reported health care use outcomes (Table 4).^13,18,19,21,22,23,25,26,28,29^ These included hospitalizations, readmissions, emergency department (ED) visits, and outpatient visits. Five SRs reported cost-related outcomes.^21,23,26,28,29^ These included costs of the intervention, total costs, cost-benefit ratios, and cost-effectiveness (eg, cost per quality-adjusted life-year, cost per PIM averted, cost per adverse drug event averted).

**Table 4.** Health Care Use Outcomes From Systematic Reviews of Studies Examining Polypharmacy Interventions

Systematic reviews	Health care use outcomes		
	Hospitalizations or readmissions	Emergency department visits	Health care costs
Johansson et al,^29^ 2016	No meta-analysis	NA	No meta-analysis
	ME	NA	NE
	Evidence quality: very low	NA	Evidence quality: very low
Thillainadesan et al,^18^ 2018	No meta-analysis	NA	NA
	NE	NA	NA
	Evidence quality: very low	NA	NA
Rankin et al,^13^ 2018	No meta-analysis	NA	NA
	ME	NA	NA
	Evidence quality: very low	NA	NA
Mizokami et al,^19^ 2019	Type I/II CMRs: RR, 1.22 (95% CI, 1.07-1.38)	NA	NA
	IE	NA	NA
	Type III CMRs: RR, 0.86 (95% CI, 0.79-0.95)	NA	NA
	DE	NA	NA
	Inpatients only: RR, 0.89 (95% CI, 0.80-0.98)	NA	NA
	DE	NA	NA
	Outpatients only: RR, 1.11 (95% CI, 0.99-1.24)	NA	NA
	NE	NA	NA
	Evidence quality: moderate	NA	NA
Lum et al,^21^ 2020	No meta-analysis^a^	No meta-analysis^a^	No meta-analysis
	DE	NE	DE
	Evidence quality: very low	Evidence quality: very low	Evidence quality: very low
Hasan Ibrahim et al,^22^ 2021	No meta-analysis		
	NE	NA	NA
	Evidence quality: very low	NA	NA
Laberge et al,^23^ 2021	No meta-analysis		No meta-analysis
	ME	NA	DE
	Evidence quality: very low	NA	Evidence quality: very low
Tasai et al,^25^2021	RR, 0.88 (95% CI, 0.78-1.00)	RR, 0.68 (95% CI, 0.48-0.96)	NA
	NE	DE	NA
	Evidence quality: low	Evidence quality: low	NA
O’Shea et al,^26^ 2022	No meta-analysis	No meta-analysis^a^	No meta-analysis
	ME	DE	DE
	Evidence quality: very low	Evidence quality: very low	Evidence quality: very low
Stötzner et al,^28^ 2022	No meta-analysis Evidence quality: very low	No meta-analysis	No meta-analysis
	ME	ME	DE
	Evidence quality: very low	Evidence quality: very low	Evidence quality: very low

Abbreviations: CMRs, comprehensive medication reviews; DE, decreased effect; IE, increased effect; ME, mixed effect; NA, not applicable; NE, null effect; RR, risk ratio.

^a^ Results are from a single study only.

#### Hospitalizations and Readmissions

Ten SRs examined hospitalizations and/or readmissions.^13,18,19,21,22,23,25,26,28,29^ One SR^19^ categorized the intensity of the intervention as low intensity (type I and type II comprehensive medication reviews [CMRs]) and high intensity (type III CMR), finding that the low-intensity intervention led to a slight increase in hospitalizations (RR, 1.22, 95% CI, 1.07-1.38) while the high-intensity intervention led to a reduction in hospitalizations (RR, 0.86; 95% CI, 0.79-0.95). This SR also examined outcomes by medication review intervention setting, finding a larger, significant outcome in inpatients (RR, 0.89; 95% CI, 0.80-0.98), compared with a nonsignificant outcome in outpatients (RR, 1.11; 95% CI, 0.99-1.24).^19^ The other SR^25^ with a meta-analysis found a null effect with regard to hospitalizations (RR, 0.88; 95% CI, 0.78-1.00). In the SRs without meta-analyses, we found mixed^13,23,26,28,29^ or null effects^22,23^; one SR without meta-analysis found a decreased effect.^21^ Four SRs examined ED visits.^21,25,26,28^ Only 1 SR^25^ conducted a pooled analysis, finding a reduction in ED visits (0.68; 95% CI, 0.48-0.96). Of the 3 other SRs, 2 relied on only 1 study each (1 found a null effect^21^ and another found a reduction in ED visits^26^), and 1 found mixed effects.^28^

#### Health Care Costs

Four SRs^21,23,26,28^ found a reduction in health care costs associated with polypharmacy interventions, and 1 SR^29^ found no significant change in nearly every study that examined this outcome. In 1 SR, estimates of the cost per quality-adjusted life-year gained ranged from £11 885 to £32 466 ($16 346 to $44 651) in the UK and Ireland.^23^ The cost per PIM avoided was estimated at €1269 (95% CI, €−1400 to €6302) ($922; 95% CI, $1017-$4582). No other SR specifically looked at quality-adjusted life-years or cost-effectiveness.

### Acceptability Among Patients and Physicians

Two SRs reported on outcomes assessing the acceptability of the intervention among patients and clinicians.^27,28^ These included acceptance or adoption of the medication-related recommendations.

The acceptability of the interventions among patients and physicians was positive among the studies reporting this outcome in one SR,^27^ while adoption rates of the interventions were found to have wide variation (16%-99%) in another SR.^28^

## Discussion

### Review of Findings

Our updated systematic overview, featuring 14 high-quality SRs comprising 179 individual studies, suggests that polypharmacy interventions show some evidence of reducing PIMs and PPOs, enhancing medication adherence, and improving medication appropriateness. However, the overall quality of evidence for these outcomes remains consistently very low. Five SRs examining mortality mostly reported no significant findings. The evidence regarding the association between polypharmacy interventions and hospitalizations or readmissions was mixed, with a tendency toward null effects. Most reviews lacking pooled effects showed either null or mixed outcomes. However, interventions among outpatients and with more intensive medication reviews were associated with reduced hospitalizations in one SR^19^ and a nonsignificant reduction in hospitalizations in another SR.^25^ Polypharmacy interventions showed limited evidence in reducing falls, with most SRs reporting no association with fall incidence or number. However, one SR reported a significant reduction in falls when PIMs were discontinued. Evidence for enhancing quality of life was consistently rated very low, with most SRs showing no significant outcomes. Moreover, despite polypharmacy being a significant risk factor for adverse drug reactions, only half of the SRs examined this outcome.

Although many SRs were of high quality according to AMSTAR 2, the evidence for most outcomes was of low to very low quality, primarily due to consistent high risk of bias. This bias often stems from the lack of blinding of participants and personnel in polypharmacy intervention studies, which is challenging unless blind tapers are used. Moreover, the evidence was downgraded in numerous reviews due to result inconsistencies, likely resulting from diverse responses among populations with various comorbidities and medications. Some studies discontinued high-risk medications, while others undertook partial changes or substitutions, potentially reducing risk but not enough to prevent significant clinical outcomes, such as mortality, hospitalizations, falls, and fractures.

An important issue that requires more research is which polypharmacy-related interventions are most effective in improving outcomes. The fact that numerous SRs had mixed findings highlights the suggestion that some interventions may be more effective than others; parsing the intensity or best components of these interventions requires further study. Only 1 SR categorized interventions into their level of intensity, creating 3 categories of CMR.^19^ A type I CMR involves a prescription review, a type II CMR involves a prescription review plus medication adherence review, and a type III CMR involves the previous categories plus a face-to-face (or video) review of medicines and conditions with the patient. The authors found that only CMR type III interventions were associated with reduced unplanned readmissions.^19^

Further study is required to understand for whom polypharmacy-related interventions prove most useful. While most SRs in our overview focused on older adult populations (age ≥65 years) with polypharmacy and multimorbidity requirements, a subanalysis by Page et al^30^suggests potentially differing outcomes within these populations. Page et al found a possibly greater reduction in mortality in patients aged 65 to 79 years compared with those aged 80 years or older, and a reduction in mortality among patients without dementia compared with those with dementia. These findings indicate a need for more studies examining subpopulations, revealing a major gap in evidence regarding the differences among specific groups might benefit more from interventions addressing polypharmacy.

### Limitations

We observed a limitation in the reviewed SRs and primary studies: insufficient detail in intervention descriptions. Inadequate information about intervention types and intensity could affect categorization and outcome assessment. To enhance reporting, journals might consider mandating the use of checklists such as the Template for Intervention Description and Replication for better intervention description.^38^ Our overview had limitations in not including Google Scholar or gray literature, potentially restricting identification of additional SRs. Although we searched 3 databases for relevant articles, our exclusion of SRs involving adults younger than 65 years limited our scope. This decision was made to encompass all adults, acknowledging the risk of polypharmacy-related adverse drug events, not only in those aged 65 years and older but also in younger adults nearing older age. Furthermore, some SRs and their studies had unclear criteria regarding the types of medications considered, whether only scheduled or also as-needed medications, over-the-counter medications, vitamins, and supplements.

## Conclusions

While the evidence base for polypharmacy-related interventions has expanded since 2019, gaps in research persist. Understanding the most useful interventions for specific high-risk populations remains a key priority. Our updated systematic overview reveals mixed findings on interventions addressing polypharmacy. They show promise in reducing potentially inappropriate medications and prescribing omissions but limited evidence in reducing mortality, hospitalizations, readmissions, or falls.

## Notes

Supplement 1. **eAppendix 1.** Full Search Strategy for Updated Systematic Overview of Systematic Reviews Evaluating Interventions Addressing Polypharmacy **eAppendix 2.** Selection Strategy **eAppendix 3.** Standardized Abstraction Form for Updated Systematic Overview of Systematic Reviews Evaluating Interventions Addressing Polypharmacy **eTable 1.** AMSTAR 2 Scores for Systematic Reviews of Studies Examining Polypharmacy Interventions **eTable 2.** Intervention Types by Systematic Review **eTable 3.** Citation Matrix
eReferences

Supplement 2.
Data Sharing Statement
