# Supplementary material for: Cumulative Update of a Systematic Overview Evaluating Interventions Addressing Polypharmacy
Source: JAMA Netw Open. 2024 Jan 10;7(1):e2350963. doi: 10.1001/jamanetworkopen.2023.50963 (PMC10782233; doi:10.1001/jamanetworkopen.2023.50963)
Supplement: Supplement 1. — eAppendix 1. Full Search Strategy for Updated Systematic Overview of Systematic Reviews Evaluating Interventions Addressing Polypharmacy eAppendix 2. Selection Strategy eAppendix 3. Standardized Abstraction Form for Updated Systematic Overview of Systematic Reviews Evaluating Interventions Addressing Polypharmacy eTable 1. AMSTAR 2 Scores for Systematic Reviews of Studies Examining Polypharmacy Interventions eTable 2. Intervention Types by Systematic Review eTable 3. Citation Matrix eReferences [file jamanetwopen-e2350963-s001.pdf]

Supplementary Online Content

Keller MS, Qureshi N, Mays A, Sarkisian C, Pevnick J. Cumulative update of a systematic overview evaluating interventions addressing polypharmacy. *JAMA Netw Open*. 2024;7(1):e2350963. doi:10.1001/jamanetworkopen.2023.50963

eAppendix 1. Full Search Strategy for Updated Systematic Overview of Systematic Reviews Evaluating Interventions Addressing Polypharmacy

eAppendix 2. Selection Strategy

eAppendix 3. Standardized Abstraction Form for Updated Systematic Overview of Systematic Reviews Evaluating Interventions Addressing Polypharmacy

eTable 1. AMSTAR2 Scores for Systematic Reviews of Studies Examining Polypharmacy Interventions

eTable 2. Intervention Types by Systematic Review

eTable 3. Citation Matrix

eReferences

This supplementary material has been provided by the authors to give readers additional information about their work.

© 2024 Keller MS et al. *JAMA Network Open.*

eAppendix 1. Full search strategy for updated systematic overview of systematic reviews evaluating interventions addressing polypharmacy

PubMed search strategy:

(("2017"[Date - Publication] : "3000"[Date - Publication]) AND english[Language])

AND

("Systematic Review"[ti] OR "Systematic Review"[tiab] OR "health technology assessment"[tiab] OR "metaanalysis"[tiab] OR "meta-analysis"[tiab] OR "Systematic Review"[Publication Type])

AND

((polypharmacy[tiab] OR deprescribing[tiab] OR deprescriptions[MeSH Terms] OR polypharmacy[MeSH Terms] OR "potentially inappropriate medication list"[MeSH Terms] OR "inappropriate prescribing"[MeSH Terms]) AND (intervention* or program* or monitor* or tool*[tiab])) AND #1 AND #2)

Cochrane database search strategy:

#1 "polypharmacy":ti,ab,kw OR "deprescribing":ti,ab,kw Publication Year from 2017 to 2022

DARE search strategy:

(polypharmacy) IN DARE FROM 2017 TO 2022

© 2024 Keller MS et al. *JAMA Network Open.*

eAppendix 2: Selection strategy

Similar to a previously published systematic overview,[1] we considered an SR to be a summary of outcomes resulting from a detailed and comprehensive plan and search strategy for relevant evidence derived *a priori*. We included SRs of studies with any study design (e.g., randomized clinical trials and observational studies) and outcome. SRs were included if they included a definition of polypharmacy or "polypharmacy" in the search criteria. We included SRs regardless of setting (e.g., inpatient, outpatient, nursing home) or population (e.g., frail older adults, patients with specific chronic diseases).

Exclusion criteria: We excluded SRs focusing exclusively on interventions implemented in low- to middle-income countries, where the average annual income level is under $14,000,[2]due to differences in care practices and healthcare infrastructure. We excluded SRs focused on antibiotic stewardship, solely about inappropriate prescribing, or medication adherence, as we felt these topics merited separate systematic overviews. Finally, we excluded SRs focused on interventions aimed at reducing or managing only one medication class (e.g., benzodiazepines, proton pump inhibitors), as we considered these to be outside of the scope of polypharmacy-related interventions. This resulted in a more focused systematic overview compared to the previously published paper.[1]

© 2024 Keller MS et al. *JAMA Network Open.*

eAppendix 3: Standardized abstraction form for updated systematic overview of systematic reviews evaluating interventions addressing polypharmacy.

Article Data	Article ID
	Abstractor
	Authors
	First Author Last Name
	Year
	Title
	Abstract
AMSTAR2 (1 or 0)	1. Did the research questions and inclusion criteria for the review include the components of PICO?
	2. Did the report of the review contain an explicit statement that the review methods were established prior to the conduct of the review and did the report justify any significant deviations from the protocol?
	3. Did the review authors explain their selection of the study designs for inclusion in the review?
	4. Did the review authors use a comprehensive literature search strategy?
	5. Did the review authors perform study selection in duplicate?
	6. Did the review authors perform data extraction in duplicate?
	7. Did the review authors provide a list of excluded studies and justify the exclusions?
	8. Did the review authors describe the included studies in adequate detail?
	9. Did the review authors use a satisfactory technique for assessing the risk of bias (RoB) in individual studies that were included in the review?
	10. Did the review authors report on the sources of funding for the studies included in the review?
	11. If meta-analysis was performed, did the review authors use appropriate methods for statistical combination of results?
	12. If meta-analysis was performed, did the review authors assess the potential impact of RoB in individual studies on the results of the meta-analysis or other evidence synthesis?
	13. Did the review authors account for RoB in primary studies when interpreting/discussing the results of the review?
	14. Did the review authors provide a satisfactory explanation for, and discussion of, any heterogeneity observed in the results of the review?
	15. If they performed quantitative synthesis did the review authors carry out an adequate investigation of publication bias (small study bias) and discuss its likely impact on the results of the review?
	16. Did the review authors report any potential sources of conflict of interest, including any funding they received for conducting the review?
	SUM AMSTAR2
Study Data	Objective
	Number included studies
	Number and types of studies (RCT and non-RCT)
	Intervention type(s)
	Description of Interventions?
	Intervention target (pharmacist, physician, patient, other?)
	Single versus multi-component interventions
	**Study (changed from patient)** population(s)
	Analyses of subpopulation(s)
	Setting(s)
	Primary outcome measure(s)
	Presence of meta-analytic techniques and any pooled estimates
	Major conclusions regarding intervention effectivenes
Outcomes by Category	(1) a positive association between intervention strategy and outcome
	(2) a negative association between intervention strategy and outcome
	(3) a null association between intervention strategy and outcome
	(4) preclusion from drawing conclusions due to limited or low-quality studies
	(5) mixed results
Quality	GRADE Score

© 2024 Keller MS et al. *JAMA Network Open.*

eTable 1. AMSTAR2 scores for systematic reviews of studies examining polypharmacy interventions.

Systematic Review Author and Date	Johansson *et al* (2016)	Page *et al* (2016)	Thillainadesan *et al* (2018)	Rankin *et al* (2018)	Mizokami *et al* (2019)	Ali *et al* (2020)	Lum *et al* (2020)	Hasan Ibrahim *et al* (2021)	Laberge *et al* (2021)	Lee *et al* (2021)	Tasai *et al* (2021)	O'Shea *et al* (2022)	Reeve *et al* (2022)	Stozner *et al* (2022)
1. Did the research questions and inclusion criteria for the review include the components of PICO?	1	1	1	1	1	1	1	1	1	1	1	1	1	1
2. Did the report of the review contain an explicit statement that the review methods were established prior to the conduct of the review and did the report justify any significant deviations from the protocol?	1	1	1	1	1	1	0	1	0	1	0	1	1	1
3. Did the review authors explain their selection of the study designs for inclusion in the review?	1	1	0	0	0	0	0	0	1	1	0	1	1	1
4. Did the review authors use a comprehensive literature search strategy?	1	1	1	1	1	1	1	1	1	1	1	1	1	1
5. Did the review authors perform study selection in duplicate?	1	1	1	1	1	0	1	1	1	1	1	1	1	1
6. Did the review authors perform data extraction in duplicate?	1	1	0	1	1	0	1	1	1	1	1	1	1	1
7. Did the review authors provide a list of excluded studies and justify the exclusions?	1	0	0	1	0	0	0	1	0	1	0	0	1	0
8. Did the review authors describe the included studies in adequate detail?	1	1	1	1	1	1	1	1	1	1	1	1	1	1
9. Did the review authors use a satisfactory technique for assessing the risk of bias (RoB) in individual studies that were included in the review?	1	1	1	1	1	1	1	1	1	1	1	1	0	1
10. Did the review authors report on the sources of funding for the studies included in the review?	0	1	0	0	0	0	0	0	0	0	0	0	0	0
11. If meta-analysis was performed, did the review authors use appropriate methods for statistical combination of results?	1	1	NA	1	1	1	NA	NA	NA	1	1	NA	NA	NA

© 2024 Keller MS et al. *JAMA Network Open.*

12. If meta-analysis was performed, did the review authors assess the potential impact of RoB in individual studies on the results of the meta-analysis or other evidence synthesis?	1	1	NA	1	0	1	NA	NA	NA	1	0	NA	NA	NA
13. Did the review authors account for RoB in primary studies when interpreting/discussing the results of the review?	1	1	1	1	0	1	0	1	0	1	0	0	0	0
14. Did the review authors provide a satisfactory explanation for, and discussion of, any heterogeneity observed in the results of the review?	1	1	1	1	1	1	0	0	0	1	1	1	1	1
15. If they performed quantitative synthesis did the review authors carry out an adequate investigation of publication bias (small study bias) and discuss its likely impact on the results of the review?	1	1	NA	1	0	0	NA	NA	NA	1	0	NA	NA	NA
16. Did the review authors report any potential sources of conflict of interest, including any funding they received for conducting the review?	1	1	1	1	1	1	1	1	1	1	1	0	1	1
SUM AMSTAR2	15	15	9	14	10	10	7	10	8	15	9	9	10	10

Notes: AMSTAR2: A MeaSurement Tool to Assess systematic Reviews

© 2024 Keller MS et al. *JAMA Network Open.*

eTable 2. Intervention types by systematic review.

Systematic Review Author and Date	Johansson *et al* (2016)	Page *et al* (2016)	Thillainadesan *et al* (2018)	Rankin *et al* (2018)	Mizokami *et al* (2019)	Ali *et al* (2020)	Lum *et al* (2020)	Hasan Ibrahim *et al* (2021)	Laberge *et al* (2021)	Lee *et al* (2021)	Tasai *et al* (2021)	O'Shea *et al* (2022)	Reeve *et al* (2022)	Stozner *et al* (2022)
Pharmacist-led medication reviews	13 studies (3 studies implemented compliance-improving strategies, 3 studies included discussions with patients about medication changes)	2 studies	4 studies	17 studies	9 (2 studies included Type I CMR, which involves a prescription review only, 3 studies included a Type II CMR, which includes a prescription review and a medication adherence review, 4 studies included a Type III CMR, which includes the Type II elements and a face-to-face review of medicines and conditions with the patient.)		5 studies (some which included patient education)	5 studies	10 studies	3 studies (studies focused on fall-risk increasing drugs)	7 studies	7 studies	5 studies	
Physician-led medication reviews	3 studies	11 studies	4 studies	5 studies						2 studies (studies focused on fall-risk increasing drugs)		2 studies	11 studies	
Nurse-led medication reviews		1 study		3 studies				2 studies (delivered in collaboration with pharmacists)						
Medication review (unspecified deliverer)						4 studies								44 studies (36 studies used a comprehensive medication review)

© 2024 Keller MS et al. *JAMA Network Open.*

Multi-disciplinary team-led interventions	(1 study included medication changes related to the patient)	4 studies	1 study			1 study	1 study			4 studies	
Geriatric assessment and screening					2 studies						
Educational programs to reduce polypharmacy	4 studies also included patient education	3 studies		4 studies included patient education, 10 studies included education to providers		1 study					
Complex, multi-faceted interventions				31 studies (including computerized decision support)							
Pharmacogenetic interventions								1 study	12 studies		
Shared decision making/patient involvement										17 studies	16 studies

© 2024 Keller MS et al. *JAMA Network Open.*

*e*Table 3. Citation matrix

Systematic Review Author and Date	Johansson *et al* (2016)	Page *et al* (2016)	Thillainadesan *et al* (2018)	Rankin *et al* (2018)	Mizokami *et al* (2019)	Ali *et al* (2020)	Lum *et al* (2020)	Hasan Ibrahim *et al* (2021)	Laberge *et al* (2021)	Lee *et al* (2021)	Tasai *et al* (2021)	O'Shea *et al* (2022)	Reeve *et al* (2022)	Stozner *et al* (2022)
Abdool 2019														X
Ailabouni 2019														X
Allard 2001	X	X												
Avorn 1992														X
Baqir 2014														X
Basger 2015				X										
Beer 2011		X												
Bernsten 2001	X										X			
Bladh 2011			X	X										
Blalock 2010						X				X				
Blenke 2018														X
Boersma 2019													X	
Boyé 2017										X				
Bregnhøj 2009	X													
Briggs 2015					X									
Brixner 2016									X			X		
Brophy 2014														
Brulhart 2011														X
Bryant 2011											X			
Bucci 2003				X										
Caffiero 2017													X	
Calbelguenne 2019														X
Campbell 1999		X				X				X				
Campins 2017				X				X	X				X	
Casper 2019							X							
Chan 2009														X
Chiarelli 2020													X	
Chiu 2018				X										
Christensen 2004														X
Claesson 1998	X													
Clyne 2015				X										
Cool 2018														X
Craig 1984														X
Crotty 2004a	X			X										X
Crotty 2004b				X										
Curtin 2020													X	
Dahl 2008														X
Dalleur 2014		X	X	X										
Davidsson 2011														X
Denneboom 2007									X					
Elliott 2017												X		
Ewan 2001														X
Finkelstein 2016a												X		
Finkelstein 2016b												X		
Finkers 2007														X
Fog 2017														X
Foubert 2020														X
Franchi 2016				X										

© 2024 Keller MS et al. *JAMA Network Open.*

Frankethal 2014	X			X		X							
Fried 2017				X			X					X	
Furniss 2000													X
Gallagher 2011		X	X	X									
García-Gollarte 2014		X		X									X
Garfinkel 2010		X											
Garfinkel 2018						X							
Gerety 1993		X											
Gillespie 2009					X								
Gillespie 2013			X										
Gillespie 2017									X				
Gnjidic 2010		X											
Granas 2019													X
Gurwitz 2008													X
Haag 2016				X									
Hahn 2018													X
Halvorsen 2010													X
Hanlon 1996	X	X		X									
Herbert 2017													X
Hernandez 2020													X
Hogg 2009								X					
Holland 2005													
Jodar-Sanchez 2015										X			
Jordan 2015													X
Keine 2019											X		
Kim 2018											X		
King 2001	X												
Köberlein-Neu 2016				X			X					X	
Komagamine 2017												X	
Komagamine 2018												X	
Kroenke 1990	X	X											
Krska 2001					X			X					
Lampela 2010	X												
Lang 2012													X
Laska 1980													X
Lee 2019											X		
Lenaghan 2007					X								
Lenaghan 2008	X												
Lenander 2014	X							X					
Lepler 1993													X
Lin 2018									X				
Lisby 2010					X								
Lowrie 2012								X					
Lupu 2017													X
Maidment 2018													X
Maidment 2020													X
Malet-Larrea 2016					X				X				
Martin Lesende 2013												X	
Massot Mesquida 2019													X
Mathys 2015													X
McCarthy 2017												X	
McDonald 2019												X	

© 2024 Keller MS et al. *JAMA Network Open.*

Messerli 2016					X									
Michalek 2014			X	X										
Milos 2013	X			X										X
Mott 2016										X				
Muir 2001		X												
Muñiz 2020														X
Muth 2016				X									X	
Muth 2018				X									X	
Naunton 2003	X													
Nishtala 2009														X
O’Brien 2018									X					
Olsson 2010	X													
Olsson 2012	X			X										
Ortega Blanco	X													
Papastergiou 2017												X		
Pasina 2016														X
Patterson 2010										X				X
Patterson 2011									X					
Petersen 2018													X	
Pitkala 2014	X	X		X										
Pope 2011	X													
Potter 2015		X												
Potter 2016						X								
Potter 2019													X	
Reynolds 2017												X		
Roberts 2001														X
Russell 2019													X	
Saldivar 2016												X		
Salisbury 2018								X						
Salonoja 2012		X				X								
San-José 2020													X	
Sano 2017														X
Scheifes 2016														X
Schmader 2004				X										
Schmidt 1998														X
Schorr 1994														X
Sellors 2003	X													
Shorr 1994														X
Siaw 2015							X							
Siaw 2017							X							
Sloeserwij 2019								X						
Smeets 2021														X
Soeresnen 2018														X
Sorensen 2004									X					
Spinewine 2007			X	X										
Sturgess 2003	X													
Sugarman 2016												X		
Tabloski 1998		X												
Tamblyn 2003				X										
Tamura 2011														X
Taylor 2003				X										
Thompson														X
Thyrian 2017				X										
Touchette 2012											X			
Trygstad 2005				X										
Trygstad 2009														
Twigg 2015									X					
van der Heijden 2019									X					

© 2024 Keller MS et al. *JAMA Network Open.*

van der Linden 2017			X	X					
van der Meer 2018						X			
van der Spek 2018									X
van der Velde 2007		X				X			
Van der Wouden 2019							X		
van Summeren 2017								X	
Verrue 2012									X
Vinks 2009	X								
Watson 2014									X
Weber 2008		X				X			
Weber 2007	X								
Wehling 2016			X	X					
Westbury 2010									X
Wilchesky 2018									X
Williams 2004	X								
Wolf 2015									X
Wouters 2017									X
Yeh 2013		X							
Zaal 2016									X
Zechmann 2019								X	
Zermansky 2006	X				X				X

© 2024 Keller MS et al. *JAMA Network Open.*

eReferences

1. Anderson LJ, Schnipper JL, Nuckols TK, et al. A systematic overview of systematic reviews evaluating interventions addressing polypharmacy. *American Journal of Health-System Pharmacy*. 2019;76(21):1777-1787.
2. The World Bank Group. World Bank Country and Lending Groups. Accessed October 20, 2023, 2023. https://datahelpdesk.worldbank.org/knowledgebase/articles/906519-world-bank-country-and-lending-groups

© 2024 Keller MS et al. *JAMA Network Open.*
