# Supplementary material for: Cumulative Update of a Systematic Overview Evaluating Interventions Addressing Polypharmacy
Source: JAMA Netw Open. 2024 Jan 10;7(1):e2350963. doi: 10.1001/jamanetworkopen.2023.50963 (PMC10782233; doi:10.1001/jamanetworkopen.2023.50963)
Supplement: Supplement 2. — Data Sharing Statement [file jamanetwopen-e2350963-s002.pdf]

Data Sharing Statement

Keller. Cumulative Update of a Systematic Overview Evaluating Interventions Addressing Polypharmacy. *JAMA Netw Open*. Published January 10, 2024.
doi:10.1001/jamanetworkopen.2023.50963

Data
Data available: No

Additional Information
Explanation for why data not available: All of the extracted data is in the manuscript tables.
